# Resolution of severe hyponatraemia is associated with improved survival in patients with cancer

**DOI:** 10.1186/s12885-015-1156-6

**Published:** 2015-03-22

**Authors:** Kirsty Balachandran, Alicia Okines, Ranga Gunapala, Daniel Morganstein, Sanjay Popat

**Affiliations:** 1 Department of Medicine, Royal Marsden NHS Foundation Trust, London, SW3 6JJ UK; 2 Department of Endocrinology, Chelsea and Westminster Hospital, London, SW10 9NH UK

**Keywords:** Cancer, Hyponatraemia, Syndrome of inappropriate antidiuretic hormone, Survival, Vasopressin-2 receptor antagonists

## Abstract

**Background:**

Hyponatraemia is a common finding in patients with cancer, and has been shown to be associated with poor prognosis in different settings. We have analysed the impact of severe hyponatraemia in patients with cancer.

**Methods:**

A retrospective review of all patients admitted to a specialist cancer hospital with a plasma sodium of less than 115 mmol/l and a diagnosis of malignancy was undertaken. Patient and tumour characteristics were analysed as well as impact of hyponatraemia management on overall survival and number of lines of cancer treatment received.

**Results:**

57 patients were identified. 84% had advanced Stage 3 or 4 cancer and approximately 85% with data available had symptoms attributable to hyponatraemia. Mean length of hospital stay was 12 days, and overall survival (OS) was 5.1 months. Plasma sodium level corrected in 56% of patients and here OS was 13.6 months compared to 16 days in those whose sodium did not correct (p < 0.001). Those whose sodium corrected were more likely to receive further lines of anti-cancer treatment.

**Conclusions:**

Severe hyponatraemia in cancer is associated with very poor survival, but correction of the sodium level leads to additional treatment and significantly greater overall survival (although it is not possible to determine if this is due to specific therapy of the hyponatraemia or the resolving hyponatraemia reflects an improvement in the clinical condition). Aggressive treatment of hyponatraemia may allow more anti-cancer treatment and improve survival.

**Electronic supplementary material:**

The online version of this article (doi:10.1186/s12885-015-1156-6) contains supplementary material, which is available to authorized users.

## Background

Hyponatraemia, defined as a serum sodium level of less than 135 mmol/L, is widely reported to be the most common electrolyte abnormality amongst hospitalised patients affecting at least 5% of inpatients [1]. Even mild hyponatraemia has been shown to be associated with increased mortality amongst inpatients [2,3]. Patients may present with a variety of signs and symptoms ranging from lethargy, nausea, headaches and potentially seizures and coma due to cerebral oedema [4]. These features are reversible on correction of the serum sodium level.

Treatment of hyponatraemia varies according to the volume status of the patient. Hypovolaemic hyponatraemia is conventionally treated with intravenous saline rehydration. When the underlying cause of hyponatraemia is Syndrome of Inappropriate Antidiuretic Hormone (SIADH), fluid restriction and in some countries, demeclocycline, urea or diuretics have been mainstays of treatment, with hypertonic saline tending to be reserved for life-threatening situations as these treatment options are often slow to instigate, poorly tolerated and ineffective [5]. However, more recent consensus statements have tended to support more aggressive treatment and the wider use of hypertonic saline [6,7]. Vasopressin-2 receptor antagonists, also known as the ‘Vaptans’, have now been licensed as a novel treatment option for SIADH [8]. These non-peptide vasopressin receptor antagonists block V2 receptors and decrease aquaporin 2 activity in the kidney, thereby reducing reabsorption and increasing diuresis of free water [9]. However their use in SIADH remains controversial, in part due to the lack of hard outcome data showing they reduce mortality [6].

SIADH is a significant cause of hyponatraemia in the setting of malignancy and is most often a paraneoplastic phenomenon due to ectopic Antidiuretic hormone (ADH) release [10]. Tumour types commonly associated with SIADH are small-cell lung cancer (SCLC), (10-15% of cases are associated with SIADH), squamous cell carcinomas of the head and neck, and lymphomas [11]. Excess release of atrial natriuretic peptide (either as a stress response or a paraneoplastic phenomenon is another possible mechanism for hyponatraemia in cancer patients [12].

Hyponatraemia is also a predictive marker of longer hospital stays and greater costs [1]. Previous studies have shown that the increased risk of mortality with hyponatraemia is also observed with hyponatraemia in cancer patients [13]. More recent data has confirmed both an increased 90-day mortality and length of hospital stay in cancer patients with hyponatraemia, recorded either on admission or during their stay [14]. In this reported dataset, although there was an increased mortality in those with mild hyponatraemia (serum sodium between 130 and 134 mmol/L) it was more pronounced with more severe hyponatraemia. The 90-day mortality was lower in those in whom the hyponatraemia improved during the admission. However, this study only included a small number of patients with severe hyponatraemia, defined as serum sodium <115 mmol/L. It remains unclear if hyponatraemia directly contributes to the increased mortality or is a marker of the severity of underlying illness. It is also unclear if hyponatraemia is associated with overall mortality, as opposed to 90-day mortality, and whether correction of hyponatraemia predicts better overall survival (OS). It is plausible, especially in patients with cancer, that correction of hyponatraemia may lead to better survival rates, for example by contributing to improved performance status and hence suitability to receive anti-cancer treatment.

In this study we have examined a large cohort of patients with severe hyponatraemia, admitted to a specialist cancer hospital. Length of hospital stay and overall survival were examined, as well as the types of malignancy, and survival was compared between those whose hyponatraemia resolved and those where it did not. This study was approved as a service evaluation by the Royal Marsden Hospital Clinical Audit Committee.

## Methods

We performed a retrospective analysis of all patients at the Royal Marsden Hospital with a confirmed malignancy and at least one episode of severe hyponatraemia (serum sodium level of <115 mmol/L) over a five-year period (1st January 2007 to 1st January 2012). Patients were identified using biochemistry databases and electronic patient records reviewed to obtain data. Fields including patient age, date of cancer diagnosis, primary tumour site, histology, stage, date of initial severe hyponatraemia, documented symptoms of hyponatraemia, date of normalisation, initial urea and creatinine level and other basic biochemistry, duration of hospital admission, admission to the intensive care unit (ICU), and date of death or last follow-up were collected. In addition data on the number of lines of anti-cancer treatment (chemotherapy, radiotherapy or surgery) were collected.

The primary endpoint of the study was to determine the median duration of hospitalisation in patients with severe hyponatraemia. Secondary endpoints included overall survival from the date of detection of severe hyponatraemia, time to normalisation, overall survival in those whose sodium did normalize compared to those who did not, and the distribution of different primary tumour sites and stage.

OS was calculated from the date of first biochemical evidence of severe hyponatraemia to date of death from any cause. Any surviving patients were censored at date of last follow-up. OS was illustrated by means of Kaplan Meier curves for the whole patient population and for stratification by sodium levels. The log-rank test was used to assess survival differences for patients in whom sodium level normalized compared with those in whom it did not. Cox regression was used to obtain hazard ratio for death. A multivariate analysis was performed using age and gender as prognostic factors. Performance status was poorly documented and therefore not included in this analysis.

Any differences in the number of patients receiving anti-cancer treatment at the time of profound hyponatraemia between the cohorts of patients with corrected and uncorrected sodium levels were assessed by Fisher’s exact test. Differences between the same cohorts for the number of lines of anti-cancer treatment received were assessed using Mann–Whitney test.

This study was exempt from Ethics committee approval at our institution and was classified as a Service Evaluation. It was approved as such by the Royal Marsden Hospital Clinical Audit Committee.

## Results

A total of 60 patients were identified with a sodium of <115 mmol/L. On review of clinical records 3 were excluded (1 due to lack of a diagnosis of malignancy, and 2 due to a high likelihood of erroneous hyponatraemia results). Of the remaining 57 patients, mean age was 60 +/− 15 (range 18–88).

The distribution of tumour primary site is shown in Table 1. The stage of malignancy at time of diagnosis of severe hyponatraemia was available in 45 patients (95%). The majority (84%) of patients had Stage 3 or 4 disease. The histological type of tumour was determined: 38.6% of all cases were adenocarcinomas, whilst 12.3% were SCLC. Surprisingly only 1 patient (1.8%) had squamous cell carcinoma.

**Table 1:** **Distribution of primary tumour site amongst patients with severe hyponatraemia**

Primary tumour site	Number	%
Gastrointestinal	18	31.7
Lung	10	17.5
Haematological	6	10.6
Breast	6	10.5
Other*	6	10.7
Urological	5	8.8
(Gynae)cological	4	7.1
Head and neck	2	3.5

*Others consists of carcinoma of unknown primary, melanoma and sarcoma.

40.4% of patients had potential symptoms recorded. 15.8% of patients were asymptomatic and the reason for admission was varied including bowel obstruction, elective retroperitoneal lymph node dissection and ureteric stenting of hydronephrosis. Admission occurred for correction of the asymptomatic hyponatraemia with fluid balance monitoring. Of those with symptoms, lethargy (14.0%), nausea-vomiting (14.0%) and confusion (10.5%), were the commonest. 50 out of 57 patients with severe hyponatraemia were admitted to hospital. The reasons for not admitting the remaining 7 patients are not recorded. Amongst those admitted, the median length of stay was 12 days +/− 15 (range 0 to 201 days). ICU admission was required in 4 patients (7%) who had admissions lasting 21, 32, 43 and 68 days respectively. Further clinical information including other biochemistry, volume status and hyponatraemia specific therapy, where available, is shown in Additional file 1.

**Figure 1:**
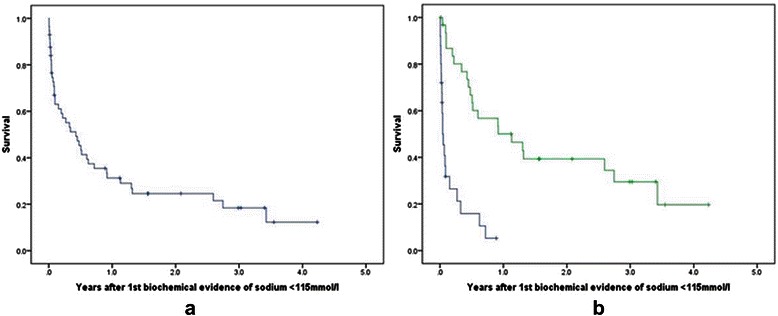
**Kaplan-Meier curves for overall survival in patients developing severe hyponatraemia. a**: Kaplan-Meier curve for overall survival in all patients developing severe hyponatraemia. **b**: Kaplan-Meier curve for overall survival in all patients developing severe hyponatraemia stratified by normalisation of sodium level.Key: Na + normalized: Green – Yes; Blue – No.

The sodium level returned to normal in 32 patients (56%). The median time to resolution of hyponatraemia was 16 days (range 0–692). OS in this cohort of patients with severe hyponatraemia was poor, at a median of 5.1 months (95% CI: 1.8 to 8.4 months, Figure 1a). In those whose sodium level returned to normal, median OS was 13.6 months (95% CI: 2.6 to 24.5 months), compared to 16 days (95% CI: 2.6 to 29.4 days) in those where it did not correct (Figure 1b, HR = 0.2 95% CI: 0.1-0.4, p < 0.001). After adjusting for age and gender in a multivariate model, normalisation of sodium remained significant (HR = 0.2 95% CI 0.1-0.3, p < 0.001).

There was no significant difference (p = 0.19) in the number of patients receiving anti-cancer treatment (chemotherapy, radiotherapy or surgery) at the time of profound hyponatraemia between the cohorts of patients with corrected and uncorrected sodium levels. More patients in those whose sodium normalised received 1 or more subsequent lines of anti-cancer therapy (median 1, range 0–3) than those in whom the Na did not normalise (median 0, range 0–1). This was significant (p < 0.001).

## Discussion

This study has examined a large cohort of patients with cancer and severe hyponatraemia (serum sodium level <115 mmol/L). We have confirmed the very poor survival in these patients, extending this to examine OS rather than the previously reported in-hospital mortality or 90 day mortality [11]. This study also confirms previous findings that resolution of hyponatraemia predicts improved survival [11]. It is not possible to determine if this reflects an effect of specific therapy for hyponatraemia or the resolving hyponatraemia reflects improvements in the underlying condition. However, the number of treatment lines received after profound hyponatraemia was significantly increased in the cohort of patients with a normalised sodium level versus those in whom it was not normalised. It is thus possible that treatment of hyponatraemia improves performance status allowing patients to receive systemic anti-cancer treatment. This may, in part, explain the dramatic differences in overall survival (13.6 months median in those whose sodium improved compared to 16 days in those who remained hyponatraemic). Given this difference in overall survival, there is an unmet clinical need for therapy to treat and potentially improve the outcome for patients with profound hyponatraemia that did not normalise.

We have also demonstrated that the vast majority of oncology patients with severe hyponatraemia have advanced cancer, with 84% having stage 3 or 4 disease. Perhaps surprisingly, nearly a third of patients had gastrointestinal tract malignancies*,* with only 17.5% having lung cancer*.* This is likely to be underpinned by several potential biases, including a greater proportion of patients overall treated for gastrointestinal cancers or increased toxicity from systemic therapy for gastrointestinal malignancies such as diarrhoea-associated hyponatraemia.

A further limitation of this retrospective study was the inability to distinguish the different causes of hyponatraemia, and it may be that if a cohort with confirmed SIADH was examined, a greater proportion would have had SCLC and head and neck tumours. Also surprisingly, the most frequent histology was adenocarcinoma, whilst only 12.3% had SCLC, and 1.8% squamous cell cancers, despite these being the histological subtypes thought to be most frequently associated with SIADH. Similarly, given the retrospective nature of the data, it is not possible to comment on the relative effects of different treatments of hyponatraemia on outcomes.

As hyponatraemia often presents with non-specific features or even in the absence of symptoms, it is possible that it is poorly recognised and this potential under-diagnosis should be considered when reviewing our data. Nevertheless this is one of the largest cohorts reported with severe hyponatraemia in cancer patients, and we have reported outcomes of OS by hyponatraemia resolution.

Historically, the treatment of hyponatraemia due to SIADH has been challenging, whilst the treatment of hypovolaemic hyponatraemia is generally straightforward, but novel treatments such as vasopressin-2 antagonists may be important in enabling treatment in patients who, prior to correction of their hyponatraemia, would not have had a performance status sufficient to permit chemotherapy. Thus, prompt correction of hyponatraemia, including the use of vasopressin-2 receptor antagonists in patients with SIADH may result in improved OS, and this is an important hypothesis that would benefit from further investigation in intervention trials.

## Conclusions

We have confirmed that severe hyponatraemia in the setting of malignancy is associated with a very poor OS and that resolution of hyponatraemia correlates with significantly better survival (although it is not possible to determine if this is due to specific therapy of the hyponatraemia) and suitability for more subsequent treatment lines compared to persistent hyponatraemia. This raises the question of whether effective treatment of the underlying hyponatraemia in patients with cancer may improve outcomes.

## Additional file

Additional file 1:
**Table showing additional clinical data on patients with severe hyponatraemia, including urea and creatinine levels at time of diagnosis of hyponatraemia, clinical assessment of volume status (where known) and hyponatraemia specific therapies (where known).**

## Abbreviations

OS: Overall survival
SIADH: Syndrome of inappropriate antidiuretic hormone
ADH: Antidiuretic hormone
SCLC: Small cell lung cancer
ICU: Intensive care unit
