# Supplementary material for: Resolution of severe hyponatraemia is associated with improved survival in patients with cancer
Source: BMC Cancer. 2015 Mar 22;15:163. doi: 10.1186/s12885-015-1156-6 (PMC4381411; doi:10.1186/s12885-015-1156-6)
Supplement: Additional file 1: — Table showing additional clinical data on patients with severe hyponatraemia, including urea and creatinine levels at time of diagnosis of hyponatraemia, clinical assessment of volume status (where known) and hyponatraemia specific therapies (where known). [file 12885_2015_1156_MOESM1_ESM.pdf]

Additional File 1: Clinical data of patients with severe hyponatraemia ($Na^+$ <115 mmol/L)

| **Urea (mmol/L)** | **Creatinine (mmol/L)** | **Primary tumour site** | **Clinical Volume status** | **Treatment of Hyponatraemia** | **Biochemistry consistent with SIADH**[1] | **Urine Na+ >40 mmol/L** |
|---|---|---|---|---|---|---|
| 2.3 | 47 | Breast | Unknown | Unknown[2] | Yes | No |
| 6.7 | 84 | Breast | Unknown | Unknown[2] | Unknown | Unknown |
| 2.0 | 42 | Lymphoma | Unknown | Unknown[2] | Unknown | Unknown |
| 5.3 | 69 | Lung | Unknown | Unknown[2] | Yes | No |
| 14.9 | 99 | Pancreas | Unknown | demeclocycline | Yes | Unknown |
| 10.8 | 143 | CUP[3] | Unknown | Unknown[2] | Yes | Unknown |
| 6.6 | 57 | Lymphoma | Unknown | Fluid restriction | Yes | Yes |
| 7.7 | 111 | endometrium | Unknown | Unknown[2] | Unknown | Unknown |
| 4.0 | 43 | Prostate | Unknown | Unknown[2] | Yes | Yes |
| 3.1 | 64 | Head and neck | Unknown | Unknown[2] | Unknown | Unknown |
| 7.7 | 9 | Breast | Unknown | None | Yes | Unknown |
| 13.8 | 100 | Ovary | Hypovolamic | IV saline | N/A | Unknown |
| 9.2 | 56 | Breast | Unknown | Unknown[2] | Yes | No |
| 3.4 | 77 | Myeloma | Unknown | Unknown[2] | Yes | No |
| 0.9 | 34 | Lung | Unknown | demeclocycline | Unknown | Unknown |
| 20.4 | 163 | Melanoma | Unknown | Unknown[2] | Yes | Unknown |
| 7.3 | 65 | Other | Unknown | Unknown[2] | Yes | Yes |
| 3.9 | 65 | Breast | Hypovolamic | Fluid restriction | N/A | No |
| 3.2 | 59 | Head and neck | Unknown | Fluid restriction | Yes | No |
| 28.9 | 203 | Colorectal | Hypovolamic | IV saline | N/A | Yes |
| 3.9 | 68 | Lymphoma | Unknown | Unknown[2] | Yes | No |
| 3.3 | 55 | Colorectal | Unknown | Unknown[2] | Unknown | Unknown |

| | | | | | | |
|---|---|---|---|---|---|---|
| 94.2 | 325 | Bladder | Unknown | Unknown[2] | Unknown | Yes |
| 6.5 | 89 | Cholangiocarcinoma | Unknown | Unknown[2] | Yes | No |
| 4.3 | 50 | Gastric | Unknown | Unknown[2] | Unknown | Unknown |
| 3.1 | 49 | Pancreas | Hypovolamic | IV saline | N/A | No |
| 27.7 | 357 | CUP[3] | Unknown | Unknown[2] | Unknown | Unknown |
| 15.6 | 99 | Colorectal | Unknown | IV saline | Unknown | Unknown |
| 4.2 | 59 | Lung | Unknown | Unknown[2] | Yes | No |
| 5.2 | 92 | Renal | Unknown | Fluid restriction | Yes | Yes |
| 9.7 | 105 | CUP[3] | Unknown | Unknown[2] | No | No |
| 2.1 | 47 | Lung | Unknown | Fluid restriction | Yes | Unknown |
| 3.6 | 40 | Lung | Unknown | Fluid restriction + demeclocycline | Yes | Unknown |
| 5.1 | 118 | Pancreas | Unknown | Unknown[2] | Yes | Yes |
| 5.0 | 53 | Pancreas | Unknown | Unknown[2] | Unknown | Unknown |
| 2.8 | 67 | Lung | Unknown | Fluid restriction + demeclocycline | Yes | No |
| 3.0 | 38 | Lung | Unknown | Unknown[2] | Unknown | Unknown |
| 7.3 | 57 | Sarcoma | Unknown | IV saline | Yes | Unknown |
| 9.3 | 53 | Colorectal | Unknown | Unknown[2] | Unknown | Unknown |
| 4.0 | 65 | Leukaemia | Unknown | Unknown[2] | Unknown | Unknown |
| 2.9 | 48 | Pancreas | Hypervolaemic | Unknown[2] | N/A | Unknown |
| 1.9 | 45 | Lung | Unknown | Fluid restriction + demeclocycline | Unknown | Unknown |
| 8.6 | 129 | Colorectal | Hypovolamic | IV saline | N/A | Unknown |
| 8.0 | 172 | Renal | Unknown | Unknown[2] | Unknown | Unknown |
| 2.0 | 40 | Lung | Unknown | IV saline | Yes | No |
| 9.7 | 60 | Oesophagus | Unknown | IV saline | Yes | No |
| 2.0 | 45 | Lung | Unknown | IV saline | Yes | No |
| 4.2 | 68 | Liver | Unknown | Unknown[2] | Yes | Yes |

| | | | | | | |
|---|---|---|---|---|---|---|
| 5.1 | 50 | Oesophagus | Unknown | Unknown[2] | Unknown | Unknown |
| 7.7 | 153 | Anus | Unknown | None | No | Unknown |
| 3.7 | 71 | Cervix | Hypervolaemic | IV saline | N/A | Unknown |
| 3.0 | 36 | Breast | Unknown | Unknown[2] | Unknown | Unknown |
| 6.4 | 90 | Lymphoma | Unknown | IV saline | No | Yes |
| 54.5 | 463 | Colorectal | Unknown | IV saline | No | No |
| 36.9 | 155 | Bladder | Hypovolamic | IV saline | N/A | Unknown |
| 15.6 | 152 | Ovary | Hypovolamic | IV saline | N/A | Yes |
| 1.3 | 64 | Gastric | Unknown | Unknown[2] | Unknown | Yes |

1. Defined as Plasma Osmolality <270mOsmol/L with Urine Osmolality >100 mOsmol/L
2. Treatment unknown but not IV saline or demeclocycline
3. Cancer of Unknown Primary
